# Recognition of seizure semiology and semiquantitative FDG‐PET analysis of anti‐LGI1 encephalitis

**DOI:** 10.1111/cns.13707

**Published:** 2021-07-22

**Authors:** Tao‐Ran Li, Yu‐Di Zhang, Qun Wang, Xiao‐Qiu Shao, Rui‐Juan Lv

**Affiliations:** ^1^ Department of Neurology, Beijing Tiantan Hospital Capital Medical University, China National Clinical Research Center for Neurological Diseases Beijing China; ^2^ Department of Neurology Xuanwu Hospital of Capital Medical University Beijing China; ^3^ Department of Neurology the Second Hospital of Hebei Medical University, Hebei Medical University Shijiazhuang China

**Keywords:** autoimmune encephalitis, FDG‐PET, LGI1, seizure semiology

## Abstract

**Aims:**

Anti‐leucine‐rich glioma‐inactivated 1 (LGI1) autoimmune encephalitis (AE) is characterized by complex manifestations of seizures. Here, we report a new seizure semiology, attempt to classify the disease by semiology type, and explore the metabolic pattern of each group.

**Methods:**

Anti‐LGI1 AE patients were retrospectively screened between May 2014 and September 2019 in our tertiary epilepsy center. All enrolled patients had seizures during long‐range video electroencephalogram (EEG) recordings, and all patients (except one) underwent [^18^F] fluoro‐2‐deoxyglucose (FDG) positron emission tomography (PET) scans. Voxel‐based metabolic analysis and z‐distribution analysis were carried out to determine the metabolic pattern.

**Results:**

Thirty‐three patients were enrolled. According to the patients’ seizure semiology, we divided the patients into four groups: focal impaired awareness seizures (FIAS, *n* = 17), faciobrachial dystonic seizures (FBDS)‐only (*n* = 6), FBDS‐plus (*n* = 8), and focal aware motor seizures (FAMS) (*n* = 2). No significant differences were found in the clinical manifestations or accessory tests except for the onset age (FIAS < FBDS‐plus) and seizure semiology. This was the first study to extensively describe the clinical manifestations and EEG of FAMS in anti‐LGI1 AE patients. In addition, we found that the patients with different semiologies all showed a wide range of abnormal metabolism, which is not limited to the temporal regions and basal ganglia, and extends far beyond our previous interpretation of FDG‐PET data.

**Conclusion:**

Our results showed that FAMS can serve as a rare indicative seizure semiology of anti‐LGI1 AE and that individuals with this disease exhibited widespread functional network alterations.

## Graphical abstract

Dozens of clinical and subclinical seizures were detected in the long‐range video EEG recording of an anti‐LGI1 encephalitis patient with focal aware motor seizures (A). Furthermore, FDG‐PET scans showed a wide range of abnormal metabolic pattern of this patient and patients with other seizure semiologies, which is not limited to the temporal regions and basal ganglia and far beyond our previous interpretation (B).

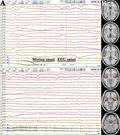

## 1 INTRODUCTION

At present, we have clearly realized that leucine‐rich glioma‐inactivated 1 (LGI1) is an extracellular component of the voltage‐gated potassium channel complex protein, which is of great importance in bridging synapses.^1^, ^2^ Autoimmune encephalitis (AE) associated with anti‐LGI1 antibodies often involves the limbic system and is characterized by symptoms of medial temporal lobe (MTL) damage (drug‐resistant epilepsy, cognitive impairment, behavioral abnormalities, etc.), sleep dysfunctions and autonomic dysfunctions.^2^ Notably, Irani et al.^3^ described a distinctive symptom termed faciobrachial dystonic seizures (FBDS) in anti‐LGI1 AE patients in detail; the authors thought the indicative seizure type frequently preceded the onset of limbic encephalitis and that initiating immunotherapy at this period can prevent disease deterioration.

Although controversy exists regarding whether FBDS are a type of seizure, recently some researchers detected generalized electroencephalogram (EEG) electrodecremental events,^4^ and others found typical focal contralateral frontal waves,^5^ both preceding the onset of muscle artifacts, indicating that FBDS were atypical seizures. In addition to this specific seizure semiology, the most common type of anti‐LGI1 AE patient is focal seizures without awareness, resembling manifestations of MTL epilepsy.^6^ Our previous study divided the seizure semiology of anti‐LGI1 AE into three types: FBDS‐only, epileptic seizures without FBDS, and FBDS plus epileptic seizures; this method of analysis revealed differences, such as ictal discharges, among the types.^7^ Other groups tried to categorize seizure types of anti‐LGI1 AE in terms of origin and consciousness^8^, ^9^; categories included the presence of FBDS, focal impaired awareness, focal aware motor/nonmotor seizures, autonomic, and generalized tonic–clonic seizures (GTCS). However, the seizure semiology of anti‐LGI1 AE has been expanding, and there is still room for improvement in the current classifications. Identifying types of unique semiology features will facilitate the early diagnosis and timely initiation of immunotherapy.

Focal aware seizures have been intensively investigated in previous studies; these seizures mostly manifest as abnormal feelings, including numbness, cold, pain, tingling, or others.^9^, ^10^, ^11^ In 2011, Andrade et al. described abnormal tonic movements involving different body regions in three anti‐LGI1 AE patients.^4^ After that, several studies also reported a similar semiology and labeled them as epileptic spasms, dystonic posture, dystonic/clonic seizures, tonic–dystonic seizures, or focal dystonia.^5^, ^10^, ^12^, ^13^, ^14^In fact, all of these symptoms represented the same entity as FBDS described by Irani et al.^3^ However, focal aware motor seizures (FAMS), independent of FBDS, have only been mentioned in two studies to the best of our knowledge, and no detailed clinical features or EEG results have been described in detail.^8^, ^15^

[^18^F]fluoro‐2‐deoxyglucose (FDG) positron emission tomography (PET) imaging plays increasingly important roles in the diagnosis, cancer screening, and follow‐up of AE. Previous studies found that FDG‐PET can significantly increase the sensitivity for detecting abnormalities in patients with AE compared with structural magnetic resonance imaging (MRI) or EEG.^16^, ^17^, ^18^, ^19^ Although considerable research efforts have been devoted to the study of AE patients’ brain networks by using multimodal MRI,^20^, ^21^ the unique nature of FDG‐PET in measuring synaptic activity is irreplaceable, as this data can reflect the impairment of the network from the perspective of metabolism.^22^, ^23^ Moreover, it is worthwhile to mention that computer‐aided brain FDG‐PET analysis further greatly improved analysis sensitivity.^24^

Here, we enrolled 33 anti‐LGI1 AE patients in our tertiary epilepsy center, and they all had seizures during the long‐range video EEG examination. According to the patients’ seizure semiology, we divided the participants into four groups: focal impaired awareness seizures (FIAS), FBDS‐only, FBDS‐plus, and FAMS. We provided a thorough description of the clinical and EEG manifestations of the two FAMS patients. In addition, by comparing matched controls, we found distinct metabolic patterns of different groups with the help of computer‐aided analysis. Since only one patient in the FAMS group underwent FDG‐PET, we performed a z‐distribution to evaluate the metabolic changes.

## 2 METHODS

### 2.1 Patient inclusion

Between May 2014 and September 2019, 57 anti‐LGI1 AE patients were retrospectively screened in our tertiary epilepsy center (Beijing Tiantan Hospital). Among them, 21 patients were excluded due to incomplete PET examination or/and negative EEG results, one patient was excluded due to incomplete clinical data, and two patients were excluded due to indistinguishable forms of attacks (limb shaking, no EEG changes). Finally, the remaining 33 patients were enrolled in our study; it should be noted that although one patient had no FDG‐PET imaging, his rare FIAS were captured by EEG recording. The diagnostic criteria of AE were based on a previous consensus,^25^ and all patients were to have positive anti‐LGI1 antibodies in their blood and/or cerebrospinal fluid (CSF) for inclusion. Patients were evaluated by at least two experienced neurologists according to a standardized protocol; this evaluation included medical history interviews, neurologic examinations, and a battery of neuropsychologic tests. In addition, comprehensive blood and CSF tests, a 3.0 T MRI scan and a 24‐h or longer EEG recording using the 10–20 system of scalp electrode placement were completed. Clinical information was obtained by reviewing the patients’ charts and databases. For the subsequent analysis of FDG‐PET, 31 healthy volunteers were recruited through advertisement. All healthy volunteers had no preexisting neurological or psychiatric illness.

### 2.2 Antibody testing

Anti‐LGI1 antibodies were confirmed with the methods described previously.^7^ Briefly, we screened the presence of brain‐reactive autoantibodies by immunostaining mouse brain sections with the patients’ serum and CSF. Then, cell‐based assays for onconeural antibodies, including anti‐Hu, Yo, Ri, CV2/CRMP5, amphiphysin, Ma2/Ta, recoverin, SOX1, titin, zic4, GAD65, and Tr (DNER), and neuronal surface antibodies, including anti‐NMDA‐R, CASPR2, AMPA1‐R, AMPA2‐R, LGI1, and GABAB‐R, were performed using a biochip test with human embryonic kidney (293) cells transfected with cDNAs encoding the relevant proteins.

### 2.3 FDG‐PET imaging acquisition and processing

Thirty‐two patients underwent FDG‐PET scans during initial clinical evaluations at diagnosis. Brain PET/computed tomography (CT) images were acquired with a hybrid PET/CT system (GE Healthcare, USA; 3‐dimensional mode, 47 image planes, 256‐mm axial field of view, 1.6‐mm transaxial resolution, and 3.3‐mm slice interval) 60 min after intravenous injection of [^18^F]‐FDG (3.7 MBq/kg). Before injection, subjects fasted for at least 6 h, and their blood glucose level was <120 mg/dl. During image acquisition, subjects rested in a quiet environment with their eyes closed. A CT scan was recorded, followed by a 15‐min emission scan consisting of three 5‐min frames. Images were reconstructed using an ordered subset expectation maximization algorithm, and vendor supplied corrections for scatter and random corrections were applied. Normal controls underwent identical scans.

Semiquantitative analysis using Statistical Parametric Mapping 12 (SPM12) (https://www.fil.ion.ucl.ac.uk/spm), implemented in MATLAB (MathWorks), was performed. FDG‐PET brain images were preprocessed, coregistered to individual T1‐weighted MRI, spatially normalized in Montreal Neurological Institute space, and normalized to global intensity with SPM12 software. Images were smoothed, and proportional scaling was used to adjust global values for metabolism. For statistical comparisons, healthy control subjects were divided into three groups (10 M/7 F, 54.8 ± 8.3 years; 6 M, 65.7 ± 2.8 years; 5 M/3 F, 62.8 ± 6.3 years) to match the number, sex ratio, and age of patients in the three different groups (FIAS, FBDS‐only, and FBDS‐plus). Images from the three groups of patients were tested for relative hypo/hypermetabolism by comparison with the corresponding reference database on a voxel‐by‐voxel basis using the general linear model by means of the SPM12 two‐sample *t*‐test design with age included as a covariate. An uncorrected threshold of *p *< 0.005 was used to compare paired groups. Only clusters of more than 50 voxels were considered.

For the only patient in the FAMS group, z‐score mapping implemented in BrainVisa software (http://brainvisa.info) was used to extract areas with differences between the patient and normal controls. Clusters of >100 voxels (8 ml) and voxels with absolute values of >1.96 z‐score (*p *< 0.05) were considered to have significantly higher metabolism than healthy controls, and these areas were extracted before z‐score maps were displayed onto anatomical images. The details are provided in our previous literature.^24^

### 2.4 Statistical analysis

Analyses were performed by using SPSS 13.0 software (SPSS). For categorical variables, Fisher's exact test was used for group comparison. For quantitative variables, the Shapiro–Wilk test indicated that the data did not conform to a normal distribution; thus, we adopted a nonparametric test (Kruskal–Wallis H test) to make group comparisons. The onset age among groups was significantly different, and we made multiple post hoc comparisons by using the “all pairwise” method. Differences were considered significant at *p *< 0.05 (two‐sided).

## 3 RESULTS

### 3.1 Clinical presentations of Anti‐LGI1 AE patients

As shown in Table 1, of the 33 anti‐LGI1 AE patients, 22 (66.7%) were male, and the median symptom onset age was 60.5 years (IQR: 58.5–68.5 years). The most common symptom was seizures (33/33, 100%), followed by cognitive impairment (27/32, 84.4%), behavioral or mood disorders (20/33, 60.6%), and sleep disorders (19/32, 59.4%). Perhaps because all the patients were enrolled from an epilepsy center, they all exhibited drug‐resistant epilepsy. Regarding the laboratory findings, 20/33 (60.6%) patients had hyponatremia (serum Na^+^<134 mmol/L); the positive rates of LGI1 antibody in blood and CSF were 100% and 93.5%, respectively; and 27/29 (93.1%) patients were LGI1‐positive in both the blood and CSF. Brain MRI scans were available for all patients before immunotherapy; specifically, 22/33 (66.7%) patients showed unilateral or bilateral abnormalities in the MTL, including hyperintensity in T2 images, swelling or atrophy, while only two (6.1%) patients showed basal ganglia hyperintensity. Notably, one patient in the FBDS‐plus group presented with abnormalities of the bilateral hippocampus and right lenticular nucleus simultaneously. Long‐range video EEG recording was also performed before immunotherapy, and patients exhibited clinical (93.9%) or subclinical (33.3%) attacks or both (27.3%). Except for one patient in the FAMS group, the remaining 32 patients all underwent FDG‐PET examination, and the average time to onset was 3.1 months; no patients had undergone immunotherapy at that time point. All of the 33 patients underwent immunotherapy: 11 received high‐dose corticosteroids, 15 received high‐dose corticosteroids combined with intravenous immunoglobulins, and seven received isolated intravenous immunoglobulins. There were 29 patients who took antiepileptic drugs; among them, 21 took one type of AED, six took two types and two took three types. All patients improved at discharge; specifically, the clinical seizures disappeared, and cognitive function improved significantly.

**TABLE 1:** General clinical features of 33 patients with anti‐LGI1 AE

	AE patients	FIAS	FBDS‐only	FBDS‐plus	FAMS	*p*‐value
No. of patients	33	17	6	8	2	NA
Onset age (y), mean (IQR)	60.5 (58.5–68.5)	55.5 (43.0–63.0)	67.0 (58.8–74.3)	68.1 (64.5–75.3)	53.5^a^	0.03^*^
M/F	22/11	10/7	6/0	5/3	1/1	0.22
Cognitive impairment	27 (84.4%)^b^	15 (88.2%)	4 (66.7%)	8 (100%)	0 (0%)^b^	0.20
Seizure semiology						
FIAS	25 (75.8%)	17 (100%)	0 (0%)	8 (100%)	0 (0%)	NA
FBDS	14 (42.4%)	0 (0%)	6 (100%)	8 (100%)	0 (0%)	NA
FAMS	2 (6.1%)	0 (0%)	0 (0%)	0 (0%)	2 (100%)	NA
FANMS	6 (18.2%)	5 (29.4%)	1 (16.7%)	0 (0%)	0 (0%)	0.23
GTCS	5 (15.2%)	4 (23.5%)	0 (0%)	1 (12.5%)	0 (0%)	0.67
Behavioral or mood disorders	20 (60.6%)	12 (70.6%)	3 (50.0%)	5 (62.5%)	0 (0%)	0.78
Sleep disorders	19 (59.4%)^b^	11 (64.7%)	2 (33.3%)	6 (75.0%)	0 (0%)^b^	0.37
Increased	15 (46.9%)^b^	8 (47.1%)	2 (33.3%)	5 (62.5%)	0 (0%)^b^	0.64
Decreased	3 (9.4%)^b^	2 (11.8%)	0 (0%)	1 (12.5%)	0 (0%)^b^	1.00
Others^f^	4 (12.5%)^b^	3 (17.6%)	0 (0%)	1 (12.5%)	0 (0%)^b^	0.79
LGI1 antibody positive						
Blood	31 (100%)^c^	15 (100%)^c^	6 (100%)	8 (100%)	2 (100%)	NA
CSF	29 (93.5%)^c^	15 (93.8%)^b^	6 (100%)	7 (100%)^b^	1 (50.0%)	1.00
Both	27 (93.1%)^e^	13 (92.9%)^d^	6 (100%)	7 (100%)^b^	1 (50.0%)	1.00
Hyponatremia^g^	20 (60.6%)^b^	10 (58.8%)	5 (83.3%)	5 (62.5%)	0 (0%)^b^	0.62
CSF hypercellularity	5 (16.7%)^d^	2 (12.5%)^b^	1 (16.7%)	2 (28.6%)^b^	0 (0%)^b^	0.80
MRI						
MTL abnormalities (B, L, R)	13, 6, 3 (39.4%, 18.2%, 9.1%)	8, 3, 2 (47.1%, 17.6%, 11.8%)	1, 1, 0 (16.7%, 16.7%, 0%)	4, 2, 1 (50.0%, 25.0%, 12.5%)	0, 0, 0 (0%, 0%, 0%)	0.11
BG abnormalities (B, L, R)	1, 0, 1 (3.0%, 0%, 3.0%)	0, 0, 0 (0%, 0%, 0%)	1, 0, 0 (16.7%, 0%, 0%)	0, 0, 1 (0%, 0%, 12.5%)	0, 0, 0 (0%, 0%, 0%)	0.20
Others^h^	10 (30.3%)	4 (23.5%)	3 (50.0%)	1 (12.5%)	2 (100%)	0.40
Long‐range video EEG						
Clinical seizures	31 (93.9%)	15 (88.2%)	6 (100%)	8 (100%)	2 (100%)	1.00
Subclinical seizures	11 (33.3%)	8 (47.1%)	0 (0%)	2 (25.0%)	1 (50.0%)	0.10
Both	9 (27.3%)	6 (35.3%)	0 (0%)	2 (25.0%)	1 (50.0%)	0.30
No. of patients who underwent PET before immunotherapy	32 (97.0%)	17 (100%)	6 (100%)	8 (100%)	1 (50.0%)	NA
Median time from symptom onset to PET (m), mean (IQR)	3.1 (1.0–4.0)	3.9 (1.3–5.0)	3.1 (0.5–6.3)	1.8 (0.8–2.0)	1.6^i^	0.19
Immunotherapy (GC, IVIG, both)	33 (11, 7, 15)	17 (6, 3, 8)	6 (3, 1, 2)	8 (1, 2, 5)	2 (1, 1, 0)	NA
AEDs (none or one type)	25 (75.8%)	12 (70.6%)	4 (66.7%)	7 (87.5%)	2 (100%)	0.64

Note

a, Patients were 68 and 39 years old, respectively; b‐e, lack of one, two, three, or four patients’ data, respectively; f, dreaminess, nightmare, somniloquy, or movements during sleep; g, hyponatremia was defined as a serum sodium concentration of less than 134 mmol/L; h, normal, senile, or nonspecific changes; i, 0.25 and 3 months, respectively. Fisher's exact test or Kruskal–Wallis H test was used for group comparisons (FIAS, FBDS‐only, and FBDS‐plus); exempt for the onset age (* means *p *< 0.05), no significant difference (*p *< 0.05) was acquired; post hoc comparisons suggested that the onset age of the FIAS group was lower than that of the FBDS‐plus group (adjusted *p *= 0.042). NA means that it was unnecessary or not possible to make comparisons.

Abbreviations: AE, autoimmune encephalitis; AEDs, antiepileptic drugs; B, bilateral; BG, basal ganglia; CSF, cerebrospinal fluid; EEG, electroencephalogram; F, female; FAMS, focal aware motor seizures; FANMS, focal aware nonmotor seizures; FBDS, faciobrachial dystonic seizures; FIAS, focal impaired awareness seizures; GTCS, generalized tonic–clonic seizures; IQR, interquartile range; L, left; LGI1, leucine‐rich glioma‐inactivated 1; M, male; MRI, magnetic resonance imaging; MTL, medial temporal lobe; NA, not available; PET, positron emission tomography; R, right.

Patients were divided into four groups according to seizure semiology, and then we made comparisons among the FIAS, FBDS‐only, and FBDS‐plus groups; however, except for the onset age, there was no significant difference in the clinical manifestations and accessory examinations. Specifically, post hoc comparisons suggest that the onset age of the FIAS group was lower than that of the FBDS‐plus group (adjusted *p *= 0.042), but this result needs to be interpreted cautiously due to the small sample size. In addition, compared with the FBDS‐only group, the MTL abnormalities and subclinical attacks of patients in both the FIAS and FBDS‐plus group showed trends of differences, but these differences did not reach statistical significance, probably due to the small sample size. The FAMS group was not included for comparison because there were only two patients in this group.

### 3.2 Typical cases with FAMS

The two patients in the FAMS group were born at term to nonconsanguineous Chinese parents, with no abnormal antenatal or postnatal issues of note, and reached developmental milestones at appropriate times. For both of these patients, the past medical history, family history of seizures, or other neurological disorders were all unremarkable.

#### 3.2.1 Patient 1

**FIGURE 1:**
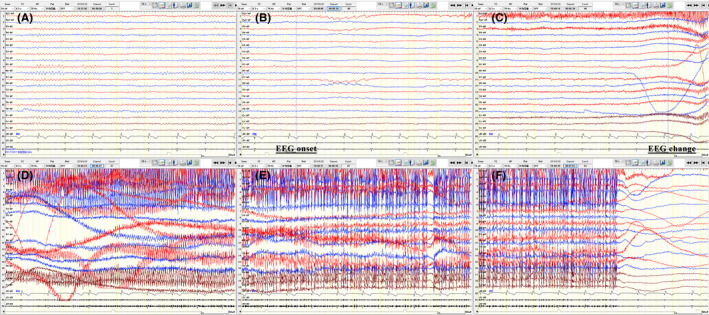
EEG performance of patient 1. A right‐handed 68‐year‐old man with anti‐LGI1 AE presented with frequent seizures, once every few minutes, in a conscious state. As shown in the long‐range video EEG data, no discharges were observed during the interictal phase (A), but dozens of rigid attacks were captured. He presented with twitches of the right eyelid and face, accompanied by a left‐sided stare and head deviation, which subsided within 35 s. Approximately 0.5 s before these attacks, the EEG signal displayed low voltage in the right frontal, central, and parietooccipital areas. Seven seconds later, a low‐amplitude fast rhythm appeared in the right central and parietal areas; then, the amplitude increased, and the frequency slowed down gradually and spread to the adjacent leads. Simultaneously, a large number of motion and electromyography artifacts were detected (B‐F, a continuous seizure process). Abbreviations: EEG, electroencephalogram; anti‐LGI1 AE, anti‐leucine‐rich glioma‐inactivated 1 autoimmune encephalitis

A 68‐year‐old man developed frequent left deviations of the head and eyes 1 week prior to evaluation, accompanied by an inability to speak, lasting tens of seconds in a conscious state. The above situation gradually deteriorated within 2 days before admission, with the frequency progressing to once every few minutes. No abnormalities were found on neurological examination, brain MRI, or chest and abdomen CT. Comprehensive onconeural and neuronal surface antibody screening showed positivity for LGI1 antibodies in both the serum and CSF at 1:100 and 1:10, respectively. In the long‐range video EEG (Figure 1), no discharges were observed during the interictal phase; nevertheless, we captured dozens of rigid attacks, presenting with twitches of the right eyelid and face, accompanied by a left‐sided stare and head deviation, which subsided within 35 s; approximately 0.5 s before these attacks, the EEG signal displayed low voltage in the right frontal, central and parietooccipital areas. Seven seconds later, a low‐amplitude fast rhythm appeared in the right central and parietal areas; then, the amplitude increased, and the frequency slowed down gradually and spread to the adjacent leads; simultaneously, a large number of motion and electromyography artifacts were detected.

#### 3.2.2 Patient 2

**FIGURE 2:**
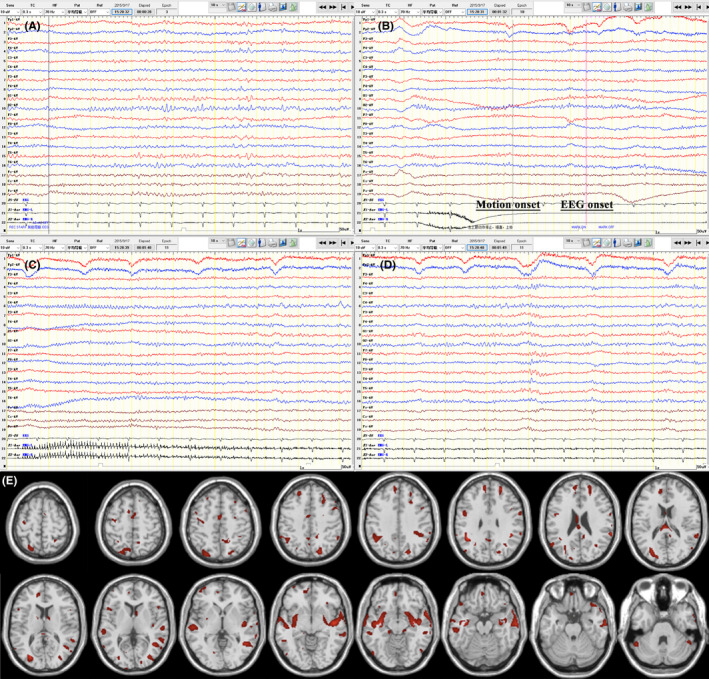
EEG performance and ^18^F‐FDG‐PET scan of patient 2. A right‐handed 39‐year‐old woman with anti‐LGI1 AE presented with rigidity, lifting, and shaking of the left upper limb in a conscious state. As shown in the long‐range video EEG recording, no discharges were observed during the interictal phase (A), but dozens of clinical and subclinical seizures were detected (B‐D, a continuous seizure process). At the beginning of seizures, there was a low‐amplitude fast rhythm in the right central areas, the amplitude gradually increased, and the rhythm returned to baseline when the limb movements stopped. During the process, the patient's left upper limb stopped suddenly, developed rigidity and slight lifting, sometimes combined with left‐sided deviation of the head. Ten to 20 s later, the left upper limb began shaking and abated in 10–40 s; she was awake throughout the process. The z‐distribution of glucose metabolism indicated hypermetabolism of many scattered brain regions, including the bilateral frontal, parietal, occipital and temporal cortex; bilateral medial temporal lobe and basal ganglia; cingulate gyrus; corpus callosum; and cerebellum (E, compared with the matched controls, voxels with absolute values of >1.96 z‐score [*p *< 0.05], clusters of >100 voxels [8 ml]). Abbreviations: EEG, electroencephalogram; ^18^F‐FDG‐PET, [^18^F]fluoro‐2‐deoxyglucose positron emission tomography; anti‐LGI1 AE, anti‐leucine‐rich glioma‐inactivated 1 autoimmune encephalitis

A 39‐year‐old woman presented with typical nocturnal GTCS 10 days prior to evaluation, which subsided within several minutes. The attack was completely controlled with an adequate dose of oxcarbazepine. However, in recent days, she had sudden, constant numbness of the left hand, followed by twitching, accompanied by flexion and rigidity of the left upper limb; these attacks occurred in a conscious state and lasted a few seconds, but occurred hundreds of times per day. Initial neurological examinations and brain MRI findings were normal. Antibody screening showed positivity for LGI1 antibodies only in the serum (1:100). Long‐range video EEG (Figure 2) detected frequent clinical and subclinical discharges; specifically, at the beginning of seizures, there was a low‐amplitude fast rhythm in the right central areas, the amplitude gradually increased, and the rhythm returned to baseline when the limb movements stopped. During the process, we found that the motions of the patient's left upper limb stopped suddenly, she exhibited rigidity and slight lifting, sometimes combined with left‐sided deviation of the head. Ten to 20 s later, the left upper limb began shaking, which abated after 10–40 s. Importantly, the patient was awake throughout the process.

Before immunotherapy, the patient underwent a brain FDG‐PET scan. We calculated the z‐distribution compared with age‐matched healthy controls to understand the metabolic changes. As shown in Figure 2, the patient showed hypermetabolism in many scattered brain regions, indicating damage to multiple brain networks.

### 3.3 Group analyses of brain glucose metabolism

In comparison to the matched reference group, FDG‐PET scans of patients in the FIAS group displayed extensive hypermetabolism in the bilateral basal ganglia, paracentral lobule, precentral gyrus, postcentral gyrus, MTL, cerebellum, lingual gyrus, insula, right superior parietal lobule, right cuneus, and left superior frontal gyrus (69–12734 voxels; peak clusters in Brodmann areas 3, 4, 6, 7, 10, 13, 17, 18, 36, and 43; Table S1 and Figure S1). In contrast, the brain areas with relatively low metabolism were mainly concentrated in the bilateral frontal cortex, parietal cortex, cingulate gyrus, and precuneus (54–9064 voxels; peak clusters in Brodmann areas 9, 19, 21, 22, 30, 31, 32, 39, 40, and 47; Table S1 and Figure S1).

In the FBDS‐only group, we observed regionally limited hypermetabolism of the bilateral cerebellum and left medial globus pallidus (154–1163 voxels; peak clusters in Brodmann areas 25; Table S2 and Figure S2) compared to the matched controls, while the left middle frontal gyrus, bilateral inferior frontal gyrus, and precuneus showed hypometabolism (56–322 voxels; peak clusters in Brodmann areas 9, 11, 31, 32, and 47; Table S2 and Figure S2).

Similar to the metabolic pattern of the FIAS group, patients in the FBDS‐plus group also presented a wide range of hypermetabolism, including the brain areas of bilateral basal ganglia, MTL, precuneus and cerebellum, left postcentral gyrus, insula and superior parietal lobule, right substantia nigra, middle occipital gyrus, and cuneus (58–6547 voxels; peak clusters in Brodmann areas 7, 13, 18, 19, 21, 38, 39, and 43; Table S3 and Figure S3). The hypometabolism regions mainly in the bilateral precuneus and right frontal cortex, small areas of the left middle frontal gyrus and posterior cingulate, right inferior parietal lobule and insula were also affected (52–2604 voxels; peak clusters in Brodmann areas 9, 10, 11, 13, 19, 30, 31, 39, 44, 46, and 47; Table S3 and Figure S3).

## 4 DISCUSSION

This study retrospectively analyzed 33 anti‐LGI1 AE patients in our tertiary epilepsy center. Seizures were observed in all patients during long‐range video EEG recordings. Then, we grouped patients according to the seizure semiology and acquired patients’ metabolic changes compared with the matched controls. This study was a larger sample study in which anti‐LGI1 patients had clinical and subclinical seizures. Importantly, we first described the clinical and EEG manifestations of two FAMS patients in detail, and we explored the metabolic pattern of each group divided by seizure semiology. All FDG‐PET scans were performed before immunotherapy due to its effect on metabolism.^18^, ^26^, ^27^

Involuntary movements have been described in up to 40%–68.8% of patients with anti‐LGI1 AE.^1^, ^5^ Despite naming differences, abnormal movements mostly belong to the FBDS entity, which is a common and characteristic manifestation of anti‐LGI1 AE.^5^, ^10^, ^12^, ^13^, ^14^ For example, authors reported tonic seizures when the tonic component was more prominent than the dystonic component.^4^ However, we thought that FAMS described in our study were different from FBDS for the following reasons. First, the FBDS were very sudden and brief, occurring with a duration of seconds,^3^, ^5^ while FAMS were relatively slow and abated after tens of seconds; and one patient had a complaint of hand numbness (patient 2). Second, FBDS entity is more of a dystonia or myotonia occurrence, while our two patients mostly presented symptoms of motor cortex stimulation, such as face twitches, head deviation, and limb shaking. Third, neither of our patients had lower limb involvement, tumbles or unconsciousness during seizures, but the incidence of these occurrences in the FBDS group could be up to 55%, 62%, and 66%, respectively.^3^, ^5^ Fourth, notably, the positive rate of scalp EEG was low during FBDS (0%–24.1%),^3^, ^7^ usually characterized as spike‐wave activity; slow, unilateral frontal waves; or generalized electrodecremental events that preceded the contralateral seizures.^3^, ^4^, ^5^, ^28^Pertinently, we detected the rhythm evolution of epileptic discharges originating from the central parietal regions of both patients. Therefore, it is important to highlight that FAMS can also serve as a rare diagnostic clue for acute encephalopathy, such as anti‐LGI1 AE. Larger sample studies are needed to support this inference.

Considerable research efforts have illustrated the limited and rigid metabolic pattern of anti‐LGI1 AE.^3^, ^7^, ^16^, ^18^, ^19^, ^26^, ^29^, ^30^, ^31^ During the FBDS stage, patients usually have hypermetabolism in the contralateral basal ganglia,^3^, ^16^, ^26^, ^30^, ^31^ sometimes accompanied by abnormal metabolism of the cortex,^5^, ^32^, ^33^ indicating complex cortical–subcortical interactions.^26^ Altered basal ganglia and MTL metabolism were found in 63%–70% and 70%–75% of cases, respectively, during the limbic encephalitis stage.^3^, ^16^, ^19^ Then, a spatiotemporal sequence was proposed by some researchers: LGI1 antibodies leak from the basal ganglia first, then from the hypothalamus, and finally from the MTL structures.^26^ However, doubts remain. First, many patients with FBDS have no abnormalities of the basal ganglia, and hypermetabolism is also not strictly contralateral.^7^, ^29^ Second, other types of AE can also have basal ganglia hypermetabolism, while the patients have no FBDS or even have no seizures.^16^, ^34^ Third, the sequence of disease progression is not stereotyped.^33^, ^35^ In addition, patients at all stages can show normal FDG‐PET metabolism^7^, ^19^ or abnormal metabolism in entirely other brain regions.^27^ These results suggest that our understanding of AE metabolism is still in its infancy, and the previous visualization method may be outdated.

Previously, we found that computer‐aided semiquantitative analysis of FDG‐PET scans could reduce the false negative rate of the MTL and basal ganglia up to 56% and 73%, respectively, compared with visual reading.^24^ By using this method on voxel‐based segmented brains, we found some surprising results that the brain areas with altered metabolism were much larger than we thought. In the FIAS group, in addition to hypermetabolism in the MTL, there was hypermetabolism of the basal ganglia, central cortex, and other regions. In the FBDS‐only group, other regions, including the cerebellum, were also hypermetabolized in addition to the basal ganglia. In the FBDS‐plus group, hypermetabolism was also not limited to the basal ganglia, MTL or central cortex. For the only patient in the FAMS group, in addition to the expected hypermetabolism in the contralateral anterior central gyrus, the z‐score indicated hypermetabolism of multiple other brain regions, including the basal ganglia and MTL. Some regions that exhibit hypermetabolism have already been reported in previous FDG‐PET studies, such as the cerebellum,^27^, ^36^ (anterior) cingulate gyrus,^27^, ^32^ thalamus,^32^ anterior central gyrus,^5^, ^27^ and occipital, prefrontal and parietal cortex.^5^, ^27^, ^36^ However, this is the first time that large area metabolic abnormalities were found in patients with the same seizure semiology, indicating that this kind of AE has caused far more damage to multiple brain networks than we know, even in the early stage of the disease. Identical conclusions were obtained in functional MRI studies.^20^, ^21^ Heine et al. found that functional connectivity alterations were not limited to the default mode network, which was closely associated with the hippocampus but also in sensorimotor, salience and higher visual networks. The impaired brain areas included the cingulate cortex, precuneus, insula, cerebellum, and medial prefrontal, frontal, temporal, occipital, parietal opercular and postcentral cortex.^20^ In addition to the aberrant functional connectivity within regions inside the MTL, other regions, such as the medial prefrontal cortex, precuneus, and posterior cingulate cluster, have also shown aberrant functional connectivity.^21^ Thus, anti‐LGI1 AE is not confined to the limbic system or basal ganglia but rather affects a wide range of brain regions and functional systems, and extralimbic symptoms and subclinical manifestations should be noted.

We acknowledge a number of limitations in this study and future directions that should be taken. First, the sample size was too small, and the patients were from a single center. Second, we found specific metabolic patterns of different groups, but it is not a simple superposition relationship; for example, the metabolic pattern of the FBDS‐plus group was not the addition of the FIAS and FBDS‐only groups; this finding is difficult to explain and may be due to individual differences, small sample sizes or different onset ages. Third, it is difficult to explain the causes of some brain regions’ abnormal metabolism, and the results cannot currently be used in individual diagnosis. Fourth, although we have corrected for the number, sex ratio, and age of the patients, some patients had intracranial ischemic changes, which could cause metabolic changes. Considering the shortcomings of our research and the limitations in this field, multicenter collaboration to include more subjects is needed to verify the repeatability of the results. Furthermore, deep‐level mechanisms, such as genetic mutations^37^ and peripheral DNA methylation,^38^ which is our future research direction, may contribute to a better understanding of our results.

## 5 CONCLUSION

Our results show that FAMS can serve as a rare indicative symptom of anti‐LGI1 AE. Furthermore, we found that patients with different seizure semiologies all showed a wide range of abnormal metabolic patterns, which is far beyond our previous interpretation of FDG‐PET data, indicating that this type of AE is associated with widespread functional network alterations. Future larger sample studies and studies from other perspectives^37^, ^38^ will contribute to the validation and interpretation of the current results.

## CONFLICT OF INTEREST

On behalf of all authors, the corresponding author confirms no conflict of interest. All authors agreed to the publication of the manuscript in its current form.

## AUTHOR CONTRIBUTIONS

RJ Lv and YD Zhang provided the clinical data. TR Li and RJ Lv acquired the literature data. Q Wang and XQ Shao polished the manuscript. TR Li drafted the manuscript. RJ Lv critically revised the manuscript for important intellectual content. All authors read and approved the final manuscript.

## Supporting information

Supplementary Material

## Data Availability

The datasets used and/or analyzed during the current study are available from the corresponding author on reasonable request.
