# Supplementary material for: Recognition of seizure semiology and semiquantitative FDG‐PET analysis of anti‐LGI1 encephalitis
Source: CNS Neurosci Ther. 2021 Jul 22;27(10):1173–81. doi: 10.1111/cns.13707 (PMC8446218; doi:10.1111/cns.13707)
Supplement: Supplementary file 1 — Supplementary Material [file CNS-27-1173-s001.docx]

**Supplementary Material**

**Recognition of Seizure Semiology and Semiquantitative FDG-PET Analysis of Anti-LGI1 Encephalitis**

**Tao-Ran Li, Yu-Di Zhang, Qun Wang, Xiao-Qiu Shao, Rui-Juan Lv***

***Corresponding author:**

Rui-Juan Lv, Department of Neurology, Beijing Tiantan Hospital, Capital Medical University, 119 South Fourth Ring West Road, Fengtai District, Beijing, 100070, PR China. Tel.: +86 10 59975052, Fax: +86 10 599785051

E-mail: [lvruijuan1981@126.com](mailto:lvruijuan1981@126.com)

**Journal Name: CNS Neuroscience & Therapeutics**

| **Supplementary Table 1, ^18^F-FDG-PET of patients in the FIAS group** | |
| --- | --- |
| **Hypermetabolism** | **Hypometabolism** |
| L. Lentiform Nucleus; L. Putamen; R. Declive of Vermis | L. Medial/Inferior Frontal Gyrus; L. Anterior Cingulate |
| (12734 voxels) | (9064 voxels in BA 9, 32, 47) |
| B. Paracentral Lobule; R. Superior Parietal Lobule | R. Precuneus; L. Posterior Cingulate; L. Cingulate Gyrus |
| (2004 voxels in BA 6, 7) | (4738 voxels in BA 30, 31) |
| R. Declive; R. Uvula; R. Lingual Gyrus | L. Angular Gyrus; L. Superior Temporal Gyrus |
| (910 voxels in BA 18) | (132 voxels in BA 22, 39) |
| R. Insula; R. Postcentral Gyrus | R. Inferior Parietal Lobule; R. Superior Temporal Gyrus |
| (301 voxels in BA 13, 43) | (103 voxels in BA 22, 40) |
| L. Insula; L. Postcentral Gyrus | R. Middle Temporal Gyrus; R. Precuneus |
| (246 voxels in BA 13, 43) | (54 voxels in BA 19, 21) |
| L. Lingual Gyrus |  |
| (227 voxels in BA 17, 18) |  |
| L. Superior Frontal Gyrus |  |
| (125 voxels in BA 10) |  |
| R. Cuneus |  |
| (97 voxels in BA 17) |  |
| L. Postcentral Gyrus; L. Precentral Gyrus |  |
| (81 voxels in BA 3, 4) |  |
| R. Precentral Gyrus |  |
| (77 voxels in BA 4) |  |
| R. Parahippocampal Gyrus |  |
| (69 voxels in BA 36) |  |

Note: Compared with the number, age, and sex matched controls, anti-LGI1 AE patients in the FIAS group showed multiple significant abnormalities of glucose metabolism in ^18^F-FDG-PET (n=17, *p*<0.005, two-sample *t*-test uncorrected for multiple comparisons and an extent threshold of 50 voxels). The locations of peak clusters were displayed in the form of brain regions and BAs.

Abbreviations: FIAS, focal impaired awareness seizures; L, left; R, right; B, bilateral; BA, Brodmann area; ^18^F-FDG-PET, [18F]fluoro-2-deoxyglucose positron emission tomography; anti-LGI1 AE, anti-leucine-rich glioma-inactivated 1 autoimmune encephalitis.

| **Supplementary Table 2, ^18^F-FDG-PET of patients in the FBDS-only group** | |
| --- | --- |
| **Hypermetabolism** | **Hypometabolism** |
| R. Cerebellar Tonsil | B. Cingulate Gyrus |
| (1163 voxels) | (322 voxels in BA 31) |
| L. Cerebellar Tonsil | L. Anterior Cingulate; L. Medial Frontal Gyrus |
| (927 voxels) | (145 voxels in BA 32, 9) |
| R. Anterior Cingulate; L. Medial Globus Pallidus | L. Inferior/Middle Frontal Gyrus |
| (154 voxels in BA 25) | (65 voxels in BA 47, 11) |
|  | R. Inferior Frontal Gyrus |
|  | (56 voxels in BA 47) |

Note: Compared with the number, age, and sex matched controls, anti-LGI1 AE patients in the FBDS-only group showed multiple significant abnormalities of glucose metabolism in ^18^F-FDG-PET (n=6, *p*<0.005, two-sample *t*-test uncorrected for multiple comparisons and an extent threshold of 50 voxels). The locations of peak clusters were displayed in the form of brain regions and BAs.

Abbreviations: FBDS, faciobrachial dystonic seizures; L, left; R, right; B, bilateral; BA, Brodmann area; ^18^F-FDG-PET, [18F]fluoro-2-deoxyglucose positron emission tomography; anti-LGI1 AE, anti-leucine-rich glioma-inactivated 1 autoimmune encephalitis.

| **Supplementary Table 3, ^18^F-FDG-PET of patients in the FBDS-plus group** | |
| --- | --- |
| **Hypermetabolism** | **Hypometabolism** |
| L. Superior Temporal Gyrus; L. Caudate Body | R. Precuneus; L. Posterior Cingulate |
| (6547 voxels in BA 38) | (2604 voxels in BA 30, 31) |
| R. Amygdala; R. Hippocampus; R. Substania Nigra | R. Middle Temporal Gyrus; R. Inferior Parietal Lobule; R. Precuneus |
| (739 voxels) | (486 voxels in BA 39, 19) |
| R. Declive of Vermis; R. Declive | L. Middle Frontal Gyrus |
| (476 voxels) | (263 voxels in BA 47, 11) |
| R. Middle Occipital Gyrus; R. Cuneus | R. Inferior Frontal Gyrus |
| (320 voxels in BA 18) | (183 voxels in BA 47) |
| L. Precuneus | R. Middle Frontal Gyrus |
| (183 voxels in BA 7) | (312 voxels in BA 10, 46, 9) |
| R. Tuber | R. Inferior Frontal Gyrus |
| (155 voxels) | (95 voxels in BA 9, 44) |
| R. Precuneus | R. Insula |
| (143 voxels in BA 7) | (82 voxels in BA 13) |
| L. Postcentral Gyrus; L. Insula | L. Medial Frontal Gyrus |
| (120 voxels in BA 43, 13) | (52 voxels in BA 10) |
| L. Middle Temporal Gyrus |  |
| (101 voxels in BA 19, 39) |  |
| L. Middle Temporal Gyrus |  |
| (84 voxels in BA 21) |  |
| L. Precuneus; L. Superior Parietal Lobule |  |
| (58 voxels in BA 7) |  |

Note: Compared with the number, age, and sex matched controls, anti-LGI1 AE patients in the FBDS-plus group showed multiple significant abnormalities of glucose metabolism in ^18^F-FDG-PET (n=8, *p*<0.005, two-sample *t*-test uncorrected for multiple comparisons and an extent threshold of 50 voxels). The locations of peak clusters were displayed in the form of brain regions and BAs.

Abbreviations: FBDS, faciobrachial dystonic seizures; L, left; R, right; BA, Brodmann area; ^18^F-FDG-PET, [18F]fluoro-2-deoxyglucose positron emission tomography; anti-LGI1 AE, anti-leucine-rich glioma-inactivated 1.


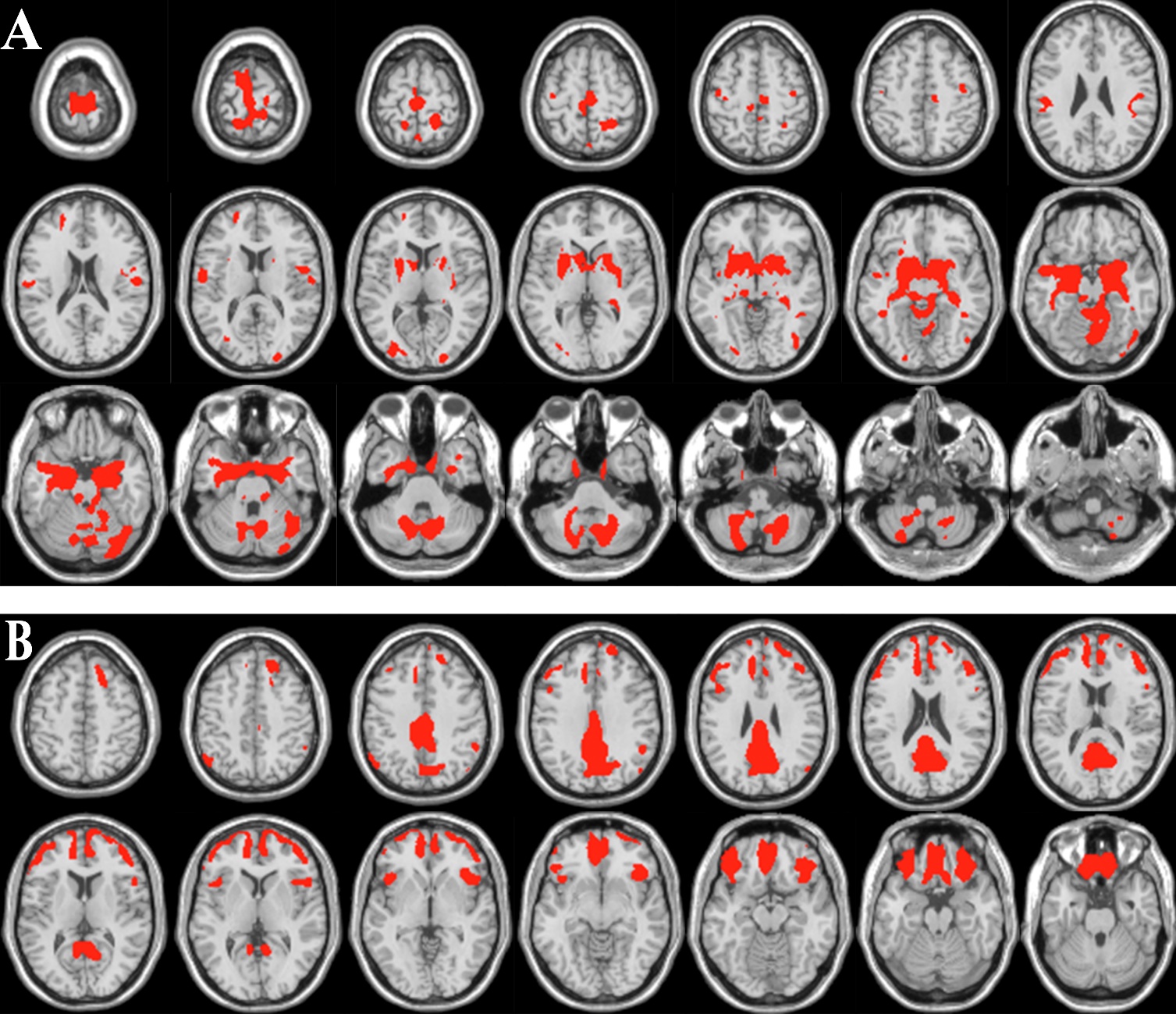


Supplementary Figure 1. ^18^F-FDG-PET of patients in the FIAS group

Group analysis by statistical parametric mapping of ^18^F-FDG-PET showed significant (*p*<0.005, two-sample *t*-test uncorrected for multiple comparisons and an extent threshold of 50 voxels) hypermetabolism **(A)** and hypometabolism **(B)** in different brain regions of investigated patients with anti-LGI1 AE patients in the FIAS group (compared with the match controls, n=17). Clusters of significant voxels projected onto magnet resonance tomograms in Montreal Neurological Institute space illustrated widespread hypermetabolism in the bilateral basal ganglia, paracentral lobule, precentral gyrus, postcentral gyrus, medial temporal lobe, cerebellum, lingual gyrus, insula, as well as right superior parietal lobule, right cuneus, and left superior frontal gyrus **(A)**, and showed hypometabolism mainly concentrated in the bilateral frontal, parietal cortex, cingulate gyrus and precuneus **(B)**.

Abbreviations: FIAS, focal impaired awareness seizures; ^18^F-FDG-PET, [18F]fluoro-2-deoxyglucose positron emission tomography; anti-LGI1 AE, anti-leucine-rich glioma-inactivated 1 autoimmune encephalitis.


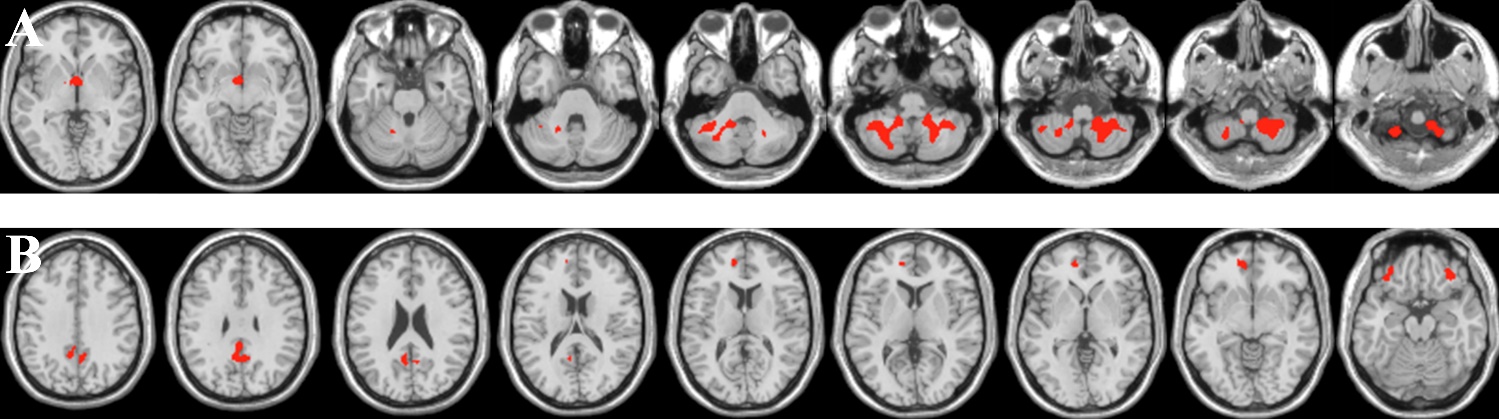


Supplementary Figure 2. ^18^F-FDG-PET of patients in the FBDS-only group

Group analysis by statistical parametric mapping of ^18^F-FDG-PET showed significant (*p*<0.005, two-sample *t*-test uncorrected for multiple comparisons and an extent threshold of 50 voxels) hypermetabolism **(A)** and hypometabolism **(B)** in different brain regions of investigated patients with anti-LGI1 AE patients from the FBDS-only group (compared with the match controls, n=6). Clusters of significant voxels projected onto magnet resonance tomograms in Montreal Neurological Institute space illustrated widespread hypermetabolism limited in the bilateral cerebellum and left medial globus pallidus **(A)**, and showed hypometabolism in the left middle frontal gyrus, bilateral inferior frontal gyrus and precuneus **(B)**.

Abbreviations: FBDS, faciobrachial dystonic seizures; ^18^F-FDG-PET, [18F]fluoro-2-deoxyglucose positron emission tomography; anti-LGI1 AE, anti-leucine-rich glioma-inactivated 1 autoimmune encephalitis.


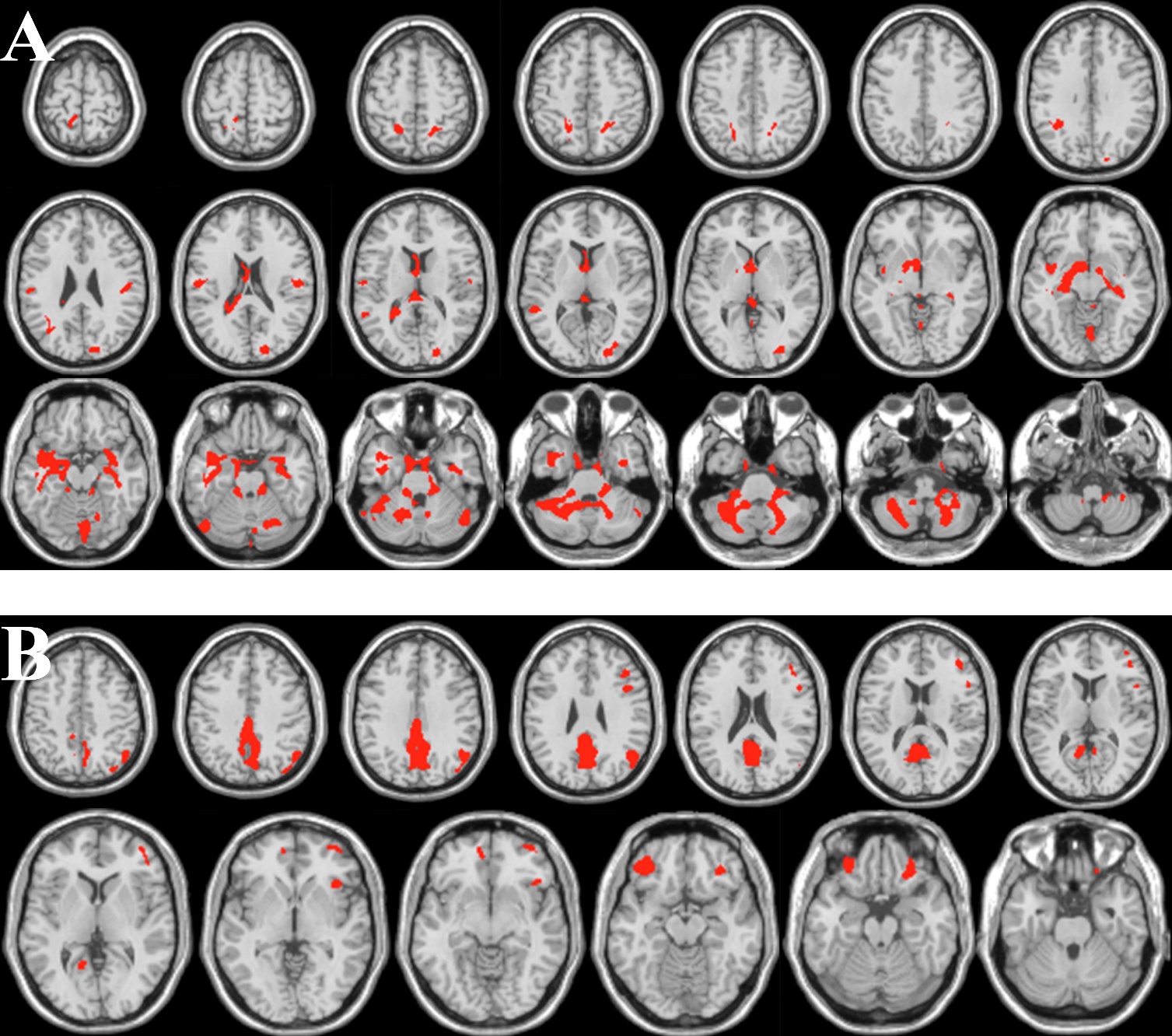


Supplementary Figure 3. ^18^F-FDG-PET of patients in the FBDS-plus group

Group analysis by statistical parametric mapping of ^18^F-FDG-PET showed significant (*p*<0.005, two-sample *t*-test uncorrected for multiple comparisons and an extent threshold of 50 voxels) hypermetabolism **(A)** and hypometabolism **(B)** in different brain regions of investigated patients with anti-LGI1 AE patients from FBDS-plus group (compared with the match controls, n=8). Clusters of significant voxels projected onto magnet resonance tomograms in Montreal Neurological Institute space illustrated widespread hypermetabolism in the bilateral basal ganglia, medial temporal lobe, precuneus and cerebellum, left postcentral gyrus, insula, and superior parietal lobule, right substania nigra, middle occipital gyrus and cuneus **(A)**, and showed hypometabolism in the bilateral precuneus, right frontal cortex, left middle frontal gyrus, posterior cingulate, right inferior parietal lobule and insula **(B)**.

Abbreviations: FBDS, faciobrachial dystonic seizures; ^18^F-FDG-PET, [18F]fluoro-2-deoxyglucose positron emission tomography; anti-LGI1 AE, anti-leucine-rich glioma-inactivated 1 autoimmune encephalitis.
